# *Epichloë* Increases Root Fungal Endophyte Richness and Alters Root Fungal Endophyte Composition in a Changing World

**DOI:** 10.3390/jof8111142

**Published:** 2022-10-28

**Authors:** Kylea R. Garces, Haley E. Sage, Natalie Christian, Sarah M. Emery

**Affiliations:** Department of Biology, University of Louisville, 139 Life Sciences Bldg, Louisville, KY 40292, USA

**Keywords:** *Epichloë*, *Ammophila*, *Fusarium*, dark septate endophytes, nitrogen deposition, global change, fertilizer

## Abstract

Plants harbor a variety of fungal symbionts both above- and belowground, yet little is known about how these fungi interact within hosts, especially in a world where resource availability is changing due to human activities. Systemic vertically transmitted endophytes such as *Epichloë* spp. may have particularly strong effects on the diversity and composition of later-colonizing symbionts such as root fungal endophytes, especially in primary successional systems. We made use of a long-term field experiment in Great Lakes sand dunes to test whether *Epichloë* colonization of the dune-building grass, *Ammophila breviligulata*, could alter fungal root endophyte species richness or community composition in host plants. We also tested whether nitrogen addition intensified the effects of *Epichlöe* on the root endophyte community. We found that *Epichloë* increased richness of root endophytes in *Ammophila* by 17% overall, but only shifted community composition of root endophytes under nitrogen-enriched conditions. These results indicate that *Epichlöe* acts as a key species within *Ammophila*, changing richness and composition of the root mycobiome and integrating above- and belowground mycobiome interactions. Further, effects of *Epichloë* on root endophyte communities were enhanced by N addition, indicating that this fungal species may become even more important in future environments.

## 1. Introduction

The plant mycobiome includes a wide variety of fungal symbionts located within the above- and belowground tissues of host plants. Shifts in the mycobiome due to biotic interactions among fungal taxa can alter host plant growth, stress tolerance, and nutrient uptake [1]. Vertically transmitted, systemic Clavicipitaceous endophytes such as *Epichlöe* spp. may have particularly strong effects on the mycobiome of their host plants, as they have priority effects within a plant host and may influence assembly of later-colonizing, horizontally transmitted fungi in both above- and belowground tissues [2]. However, effects of *Epichlöe* on the plant mycobiome are not consistent. For example, *Epichlöe* has been shown both to reduce [3] and increase colonization of roots by arbuscular mycorrhizal fungi (AMF) [4,5,6,7]. *Epichlöe* has also had mixed effects on leaf endophytes in a variety of host plant species [8,9,10]. *Epichlöe* effects on root endophyte (non-AMF) communities are not well-understood, but seem minimal in the few studies that have examined them [11,12,13]. However, the magnitude of the effect of *Epichlöe* on mycobiome composition may depend on environmental conditions and resource availability (e.g., [14,15]), both of which are shifting in the Anthropocene [16,17].

Anthropogenic nitrogen (N) enrichment may be a particularly influential aspect of global change on fungal interactions and mycobiome community composition, as resource availability directly impacts plant-fungal associations [18]. Activities associated with agriculture, industry, wastewater, and fossil fuel combustion has more than doubled rates of nitrogen input into the terrestrial nitrogen cycle [19], and atmospheric N deposition is expected to increase globally by 250% over the next century [19,20]. Nitrogen enrichment is known to directly influence mycobiome composition within plant organs, for example by reducing root mycobiome diversity [21,22,23]. Aboveground, N addition is associated with decreased foliar endophyte diversity [24,25]. The few studies that have explored N enrichment effects on *Epichlöe* showed that N addition enhanced *Epichlöe* benefits to hosts [16,26]. For example, increasing soil N improved the alleviation of drought stress provided by endophytes to some grasses [27]. However, it is unclear whether N availability alters above-belowground fungal interactions within host plants.

Changes in N availability may be particularly impactful on mycobiomes of plants in low nutrient primary successional ecosystems such as Great Lakes sand dunes. The U.S. Great Lakes coastal region has high N enrichment due to agricultural, atmospheric, and point-source inputs [28]. For example, atmospheric N deposition concentration levels, especially of NH_4_^+^, into Great Lakes ecosystems have increased 400% from historic levels [29,30] while dissolved inorganic N in Great Lakes coastal wetlands has risen as a direct result of row-crop agriculture in the region [28]. The dominant dune-building grass in this region, *Ammophila breviligulata* (hereafter *Ammophila*) harbors a variety of fungal symbionts including the systemic endophyte, *Epichlöe amarillans* (hereafter *Epichlöe*) [31], which is found in approximately one third of natural *Ammophila* populations in the Great Lakes [32,33,34,35] and in almost all nursery stock used for dune restoration work [36]. This provides an ideal system to examine effects of *Epichlöe* and N enrichment on other aspects of the plant mycobiome.

Here, we evaluated the effects of *Epichlöe* and N addition on community composition of fungal root endophytes associated with *Ammophila* in a long-term experiment within the Great Lakes dunes. Specifically, we asked: Does colonization of the host grass by *Epichlöe* alter root fungal endophyte species richness or community composition? Furthermore, if so, does N addition intensify the effects of *Epichlöe* on the root endophyte community? While very little is known about how *Epichlöe* interacts with root endophyte communities in general, earlier work in this dune system showed that *Epichlöe* reduced diversity of other root-associated fungal communities (AMF) in *Ammophila* [14]. AMF and non-mycorrhizal root endophyte communities often show opposing responses to changing conditions [37,38,39,40] (although also see [41]), and so we expected that root endophyte richness would increase in response to *Epichlöe presence*. By enhancing the plant-*Epichlöe* symbiosis, we also expected that N addition would strengthen the effects of *Epichlöe* on root endophyte diversity and composition. Alternatively, N enrichment could act as an environmental filter that limits which fungal species can colonize plant hosts, or could enhance dominance of certain taxa at the expense of mycobiome biodiversity, independent of *Epichlöe* [21,22,23,42]. Our findings provide some of the first evidence that *Epichlöe* can increase root endophyte richness in host plants, and that nitrogen enrichment strengthens the effects of *Epichlöe* on root endophyte community composition.

## 2. Methods

### 2.1. Experimental Design

In May 2010, we established a factorial field experiment on a bare dune blowout approximately 200 m from the shoreline of Lake Michigan in Leelanau State Park, Michigan, USA (45.8109640 N, 85.8345780 W). To manipulate the presence or absence of *Epichloë* in *Ammophila*, we used endophyte-free seeds collected from nearby dunes, germinated seedlings of *Ammophila* in the lab, and either artificially inoculated seedlings with *Epichloë* (E+) or sham inoculated them with sterile water (E−) (see details in [34]). Plants were clonally propagated in a greenhouse and then transported to the field. Into 90 2 m × 2 m plots, we transplanted 25 E+ or 25 E− *Ammophila* plants and monitored plots yearly from 2010–2020. Because plants can sometimes lose *Epichloë* symbionts over time [35,43], we used commercial immunoblot kits (Phytoscreen: Agrinostics, Watkinsville, GA, USA) to assess treatment reliability in two tillers per plot in 2019. Any inconclusive assay results were followed up with microscopy to confirm presence or absence of hyphae within leaf tissue. 91% of E+ treatment plot tillers maintained evidence of *Epichloë* infection, while 89% of E− plot tillers still lacked *Epichloë*.

In 2016 we introduced N addition treatments to a subset of 60 plots in the long-term experiment (30 E+ and 30 E− plots). One third of plots (10 each E+ and E−) received a low level of N (0.5 g NH_4_^+^ m^−2^), corresponding to N deposition rates in the nearby urban centers of the Midwest [30], another third received high levels of N (10 g NH_4_^+^ m^−2^), comparable to global N addition experiments [44], and the last set of plots received no added fertilizer (control). We added N as urea slow-release fertilizer (ESN Urea coated fertilizer: Nutrient Ltd., New Madrid MO, USA), applied twice yearly: once in May at the beginning of the growing season, and again mid-season in July. Due to COVID-19 travel restrictions, we were unable to apply fertilizer in May 2020, but treatments resumed as planned in July 2020.

### 2.2. Root Endophyte Isolation & Identification

In July 2020, we excavated roots from 2 randomly chosen visibly healthy *Ammophila* tiller clumps per plot in the N addition experiment (n = 60 plots). These tillers with attached roots were kept at 4 °C and transported on ice back to the lab at the University of Louisville for processing. One root segment per tiller was cut from each *Ammophila* plant and surface-sterilized using standard methods. In brief, roots were soaked in 95% ethanol for one minute then in a 1% bleach solution for two minutes. Root samples were then washed with 70% ethanol for two minutes, then rinsed with autoclaved water 3 times. After surface-sterilization of each plant root, five 3–5 mm root segments were chosen haphazardly and plated tip first into Petri dishes containing 2% malt extract agar (MEA) + 10 mL Penicillin-Streptomycin to decrease risk of bacterial contamination. Plates were then sealed with parafilm and stored in a cabinet at room temperature. Plates were monitored for approximately two weeks for fungal growth. A subset of plates was stored at 4 °C to reduce spread of fast-growing endophytes and encourage growth of slower-emerging fungi. There were no differences in taxa seen between room temperature vs. refrigerated plates, so room temperature fungal cultures were used for this study. Emergent hyphae were sub-cultured onto new MEA plates to isolate individual taxa. Isolates were allowed to grow until the colony covered the agar plate. Isolates were then grouped into morphotypes based on color and mycelial growth patterns. Once morphotypes were determined, at least one isolate from each morphotype group was identified using Sanger sequencing of the fungal ITS region (see below). For common morphotypes, multiple representative fungal isolates were sequenced. Fungal isolates were also vouchered in sterile water for long-term storage.

To verify that our morphotype designations were accurately grouped and to taxonomically identify fungal isolates, we extracted DNA and conducted polymerase chain reaction (PCR) using Sigma Aldrich Extract-N-Amp™ Plant Tissue PCR Kits [45]. We used ITS1F and LR3 primers to amplify the nuclear ribosomal internal transcribed spacer region and 5.8S gene (ITSrDNA) and approximately 600 bp of the adjacent large ribosomal subunit (LSUrDNA) [46]. Each 20 μL reaction included 4 mL molecular-grade water, 10 µL PCR Ready Mix (includes buffer, salts, dNTPs, and Taq polymerase), 1 µL ITS1F primer, 1 µL LR3 primer, and 4 µL template DNA. Amplification was done using BioRad T100 Thermal Cycler. The thermocycler settings were conducted as follows: initial three minutes of denaturation at 95 °C, followed by 37 cycles (30 s of denaturation at 95 °C, 30 s of annealing at 55 °C, and two minutes of elongation at 72 °C. and 10 min final extension at 72 °C). To confirm successful gene amplification, gel electrophoresis using SYBR Safe (Invitrogen) produced single bands for all products as well as no bands for negative controls. PCR products were enzymatically cleaned using Illustra ExoProStar (Cytiva Life Sciences) following the manufacturer’s protocol. Purified PCR samples were then sent to Eurofins Genomics (Louisville, Kentucky) for Sanger sequencing of both forward (ITS1F) and reverse (LR3) reads.

We used Sequencher v. 5.4 (Gene Codes Corporation, Ann Arbor, MI, USA) to assemble sequences into operational taxonomic units (OTUs) according to 97% ITS sequence similarity, with a minimum of 40% overlap. We used the NCBI Basic Local Alignment Search Tool (BLAST) [47] and the Ribosomal Database Project (RDP) Bayesian Classifier with both the Warcup [48] and UNITE [48] ITS training sets to obtain best match taxonomic names for each OTU (Table S1). Sequence data are archived at GenBank under accession numbers OP679885-OP679925.

### 2.3. Statistical Analysis

To analyze shifts in root fungal endophyte species richness in response to *Epichloë* presence and N addition, we conducted treatment level comparisons using a general linear mixed-effects model, with nitrogen addition treatment, *Epichloë* presence and their interaction as fixed effects and spatial plot level factors (row and column) as random effects. Neither row, column, nor their interactions with plot level factors were significant (data not shown), so they were removed from the model for subsequent analyses to better identify main treatment effects. These analyses were performed using R version 1.4.1106.

We used a two-factor PERMANOVA to analyze changes in root fungal endophyte community composition [49], with N addition treatment and *Epichloë* presence or absence, and the interaction between the two treatments as fixed effects. Using a binary community matrix comprised of presence and absence of OTUs, we performed a PERMANOVA using the Bray–Curtis distance metric with 9999 permutations under a reduced model with Type III sum of squares. Singleton taxa were excluded from this analysis to improve resolution. Heterogeneity of the community in response to the different treatment factors was tested using PERMDISP [50]. To visualize differences in root endophyte communities, we then conducted a nonmetric multidimensional scaling (NMDS) ordination [51] with Bray–Curtis dissimilarity measures. Finally, a SIMPER (similarity percentages species contribution) analysis was used to determine which fungal species contributed most to differences in community composition among treatment combinations [52]. The PERMANOVA and NMDS ordination were conducted in R using the VEGAN package [53]. PERMDISP and SIMPER analyses were conducted in Primer v. 6 [54].

## 3. Results

**Figure 1:**
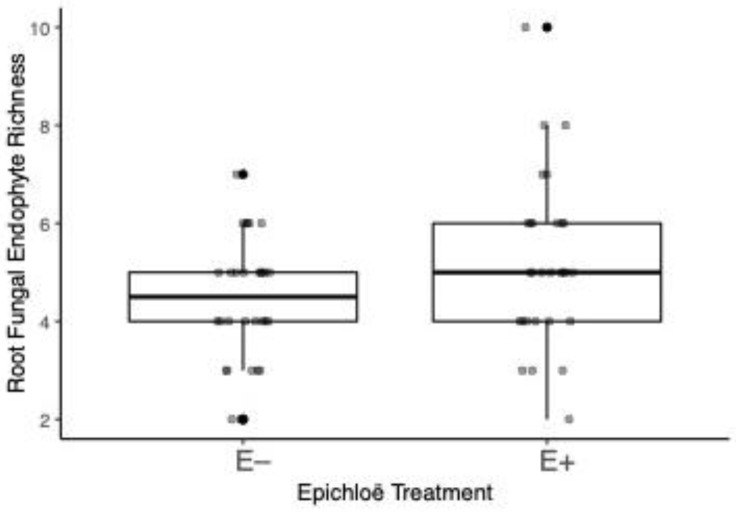
Root fungal endophyte species richness per plot from *Ammophila* plants where *Epichloë* was either present or absent. Treatment medians and quartiles shown, with points jittered to aid with viewing. Darker circles indicate overlapping data points.

We identified a total of 23 OTUs and 11 singleton taxa in our study (Figure S1). Root endophyte species richness increased by 17% when *Epichloë* was present within the host grass (Table 1, Figure 1). However, N addition had no significant main or interactive effects on root endophyte richness (Table 1).

**Figure 2:**
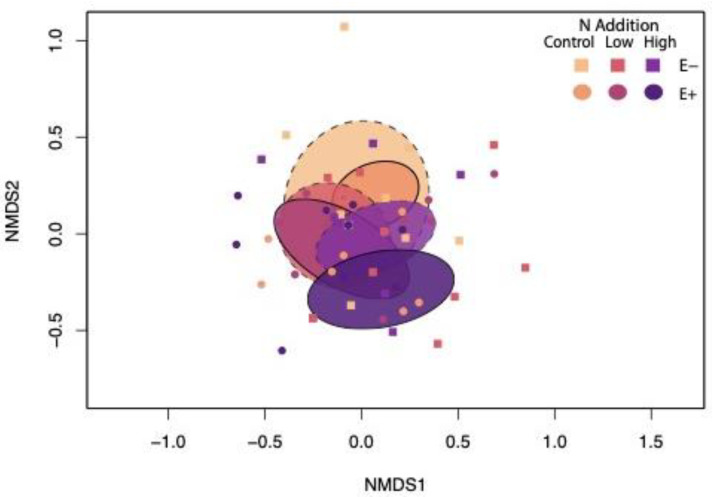
Nonmetric multidimensional scaling plot displaying differences in root endophyte community composition among *Epichloë* and N addition treatments. 2D stress = 0.2. Ellipses represent standard deviation of point scores for each treatment interaction. Dashed lines represent E− plots.

In contrast, root endophyte community composition was affected by N addition, especially where *Epichloë* was present, as indicated by the presence or absence of overlap in treatments within the ordination (Table 1, Figure 2). Pairwise comparisons showed significant differences between all N treatments for E+ plants, and no significant differences between N treatments for E− plants (Table S2).

*Epichloë* and N addition also interacted to affect heterogeneity among root endophyte communities within treatments, as shown by the PERMDISP analysis (Table 1) and NMDS ordination, where decrease in oval size corresponds to decreased heterogeneity (Figure 2). Pairwise contrasts (Table S2) indicated that low and high levels of N addition reduced root endophyte community heterogeneity in *Ammophila* roots by 32–36% compared to plants in the control treatment, but only when *Epichloë* was absent. N addition had no effects on heterogeneity when *Epichloë* was present. In control N treatments, *Epichloë* presence reduced heterogeneity of root endophyte communities by 43% compared to plants lacking *Epichloë*.

Finally, The SIMPER analysis indicated that two taxa, *Microdochium bolleyi* and *Fusarium* sp., were common taxa within root endophyte communities across all treatments (Table S3). *Fusarium* sp., along with *Leptosphaeria* sp., *Cadophora* sp., and *Sarocladium strictum* showed reduced occurrences in response to N addition, while a different *Fusarium* sp. and *Acremonium* sp. increased in frequency in response to N addition (Table S4).

## 4. Discussion

*Epichloë* influenced belowground mycobiome diversity and composition in this dune grass system, especially under N-enriched conditions, providing moderate evidence that *Epichloë* may act as a key species restructuring the above- and belowground mycobiome of host plants. Several potential explanations exist for why root fungal endophyte communities increased in richness in response to *Epichloë* infection. It might be expected that *Epichloë* would suppress root endophyte diversity due to its ability to produce systemic alkaloids [55,56,57] which directly inhibit growth of other fungal species, including pathogens [4,5,57,58,59,60]. However, indirect effects of *Epichloë* on its plant host may override direct mycobiome interactions to improve conditions for root endophyte communities. Within the harsh sand dune environment specific to our study, *Epichloë* acts as a mutualist, increasing *Ammophila* survival, vegetative growth, and belowground biomass [14,34,61]. In other systems, *Epichloë* can improve rhizosphere characteristics including soil fertility, root morphology, soil nutrients, and organic carbon [62,63]. By increasing habitat space in roots and improving belowground conditions, *Epichloë* may indirectly facilitate root endophyte diversity. The other studies that have demonstrated no effects of *Epichloë* on root endophyte communities were conducted in less extreme habitats such as agricultural fields and prairies [11,12], where indirect effects of *Epichloë* on host plants may be less important.

*Epichloë* infection also altered root endophyte community composition in *Ammophila*, especially under N-enriched conditions; while under ambient conditions, the presence of *Epichloë* caused root fungal endophyte communities to converge across plots. These findings suggest that *Epichloë* acts a filter to restructure the endophyte communities that colonize *Ammophila* roots, possibly by altering the physical or chemical environment of host plants, such as root exudate chemistry [64,65]. Plant mycobiome composition can be strongly influenced by plant secondary metabolites [66,67], and *Epichloë* is known for enhancing alkaloid production inside host plants [68]. Alkaloids are nitrogen-rich secondary metabolites, so adding nitrogen could alter *Epichloë’s* ability to produce these chemicals within hosts, either positively [69,70,71] or negatively [72] (but see [73] where no effect was found), which could induce a strong filter on root endophyte species composition.

We were able to identify several fungal taxa responsible for the shifts in root endophyte community composition. Both *Microdochium bolleyi* and *Fusarium* sp. were common taxa within root endophyte communities across all treatments. This *Fusarium* sp. best matched with *Fusarium fujikuroi* in the BLAST database (99.82% match), though we recognize that species designations within *Fusarium* usually require a tef1 sequence, which we did not have. *Microdochium bolleyi* is a common dark septate endophyte, primarily of grasses [74,75,76], including dune grasses of the Pacific Northwest [77]. A recent study found no effects of *Epichloë* exudates on *M. bolleyi* growth in an in vitro assay [6], which our field results support. However, *F. fujikuroi* (putative) was suppressed by *Epichloë* presence in N-enriched conditions, along with *Sarocladium strictum*, *Leptosphaeria* sp., and *Cadophora* sp. While the functions of many root endophytes are unknown, *Fusarium fujikuroi* has been classified in other systems as an asymptomatic nonobligate root symbiont best known for its gibberellin production [78,79] and has been found in other marine and coastal systems, along with *S. strictum* [80]. Since *Epichloë* is known to stimulate gibberellin production in both seeds and plants [81], any benefits that *F. fujikuroi* provides to hosts may become redundant. *Sarocladium*, *Leptosphaeria* and *Cadophora* are common root endophyte genera [82,83] with functions ranging from commensal to parasitic to saprophytic, making generalizations difficult.

Two taxa increased in abundance in response to N addition for *Epichloë* colonized plants: *Fusarium sporotrichioides* (putative) and *Acremonium* sp. *Fusarium sporotrichioides* is a known pathogen of maize and an opportunistic pathogen of other cereal crops [84,85]. In agricultural systems, N fertilization often increases abundance of this and related *Fusarium* spp., possibly due to changes in plant N-metabolism (e.g., [86]). This may explain why increased N availability increased the occurrence of this species in *Ammophila* roots. *Acremonium* species may act as potential mutualists by increasing root growth in host plants [87], and so may hold a functionally similar role to *Epichloë*. While very little is known about root endophyte biology, especially in non-agricultural systems, these species-specific responses provide some insights into how *Epichloë* may filter mycobiome communities.

In conclusion, by manipulating the presence of *Epichloë* in a long-term experiment, we found moderate evidence that this systemic endophyte is acting as a key species within *Ammophila*, changing diversity and composition of the root mycobiome and integrating above- and belowground mycobiome interactions. Further, effects of *Epichloë* on root endophyte communities were enhanced by N addition, indicating that this fungal species may become even more important in future environments. *Ammophila* is widely used in coastal dune restorations, and the importance of plant-fungal symbioses for restoration efforts are starting to be recognized [88]. Future work should address the consequences of such shifts in mycobiome communities for host plants, as intentional manipulation of mycobiome interactions may improve future restoration efforts in changing environments.

## Figures and Tables

**Table 1:** Statistical results from analyses of root fungal endophyte species richness (GLM), community composition (PERMANOVA), and community heterogeneity (PERMDISP). Statistically significant results are indicated in bold and by an asterisk (* *p* < 0.10, ** *p* < 0.05).

	Richness		Composition		Heterogeneity	
Factor	F	*p*	Pseudo-F	*p*	Pseudo-F	*p*
*Epichloë*	3.71	0.06 *	0.96	0.43	0.19	0.71
N addition	0.51	0.60	2.57	0.01 **	0.68	0.57
*Epichloë* × N add	0.35	0.71	1.80	0.06 *	2.52	0.07 *

## Data Availability

Data are available upon request from the authors.

## Supplementary Materials

The following supporting information can be downloaded at: https://www.mdpi.com/article/10.3390/jof8111142/s1, Table S1. Comparison of different fungal databases (UNITE, Warcup, BLAST) to identify fungal root endophyte OTUs detected within the study to taxon. Percentages listed in the table are indicative of close matches for designated taxa. For UNITE and Warcup, the percentages given in brackets indicates confidence thresholds whereas BLAST displays the percent identity compared to its best match. OTUs beginning with S indicate singleton taxa. Table S2. Pairwise comparisons of community composition (PERMANOVA) and heterogeneity (PERMDISP) between N-addition treatments within each *Epichloë* treatment. Table S3. Similarity Percentages (SIMPER) Analysis results, showing taxon contributions to community composition similarity within each *Epichloë* × N addition treatment, up to 90% cumulative composition. Table S4. Similarity Percentages (SIMPER) Analysis results, showing root endophyte taxon average frequencies and their contribution to community differences among N addition treatments when *Epichloë* was present. Figure S1. Heatmap displaying presence/absence of fungal root endophyte OTUs within each *Epichloë* and N-addition treatment.
