# Supplementary material for: Epichloë Increases Root Fungal Endophyte Richness and Alters Root Fungal Endophyte Composition in a Changing World
Source: J Fungi (Basel). 2022 Oct 28;8(11):1142. doi: 10.3390/jof8111142 (PMC9697489; doi:10.3390/jof8111142)

Supplementary Material

Title: *Epichloë* increases root fungal endophyte richness and alters root fungal endophyte composition in a changing world

Authors: Kylea R. Garces, Haley E. Sage, Natalie Christian, and Sarah M. Emery

Table S1. Comparison of different fungal databases (UNITE, Warcup, BLAST) to identify fungal root endophyte OTUs detected within the study to taxon. Percentages listed in the table are indicative of close matches for designated taxa. For UNITE and Warcup, the percentages given in brackets indicates confidence thresholds whereas BLAST displays the percent identity compared to its best match. OTUs beginning with S indicate singleton taxa.

OTU	Unite	Warcup	BLAST
1	*Exophiala salmonis* [90%]	*Exophiala opportunistica* [65%]	*Exophiala equina* [96.14%]
2	*Exophiala equina* [100%]	*Exophiala equina* [45%]	*Exophiala equina* [99.15%]
3	*Rhexocercosporidium panacis* [46%]	*Leveillula lanuginose* [51%]	*Cadophora sp.* [99.40%]
6	*Penicillium angulare* [83%]	*Penicillium bilaiae* [72%]	*Penicillium bilaiae* [99.91%]
7	*Acremonium* sp. [100%]	*Fusarium* sp FAN2011a [89%]	Unk. *Acremonium* clone [99.82%]
8	*Stachybotrys elegans* *[*100%]	*Fusarium* sp FAN2011a [84%]	*Stachybotrys bisbyi* [99.82%]
10	*Arthrinium malaysianum* [95%]	*Nigrospora* sp5 MU2012 [93%]	*Arthrinium sacchari* [99.91%]
12	*Sarocladium strictum* *[*94%]	*Fusarium* sp FAN2011a [81%]	*Sarocladium strictum* [99.38%]
13	*Aspergillus fumisynnematus* [100%]	*Aspergillus fumigatus* [82%]	*Aspergillus fumigatus* [99.56%]
15	*Talaromyces flavus* [97%]	*Penicillium funiculosum* [71%]	*Talaromyces amestolkiae* [100%]
16	Ascomycota sp [79%]	*Alternaria* sp3 MU2012 [93%]	*Leptosphaeria sp.* [99.91%]

18	*Fusarium udum* [96%]	*Fusarium* sp FAN2011e [68%]	*Fusarium fujikuroi* [99.82%]
31	Xylariales sp [100%]	*Pestalotiopsis* sp7 MU2012 [62%]	*Microdochium bolleyi* [99.73%]
46	*Alternaria* sp3 MU2012 [91%]	*Alternaria* sp3 MU2012 [100%]	*Alternaria alternata* [99.91%]
55	Xylariales sp [100%]	*Pestalotiopsis* sp7 MU2012 [65%]	*Gloeocercospora sorghi* [96.82%]
57	Nectriaceae sp [100%]	*Fusarium* sp FAN2011a [69%]	*Fusarium sporotrichioides* [99.73%]
61	*Elaphocordyceps capitata* [18%]	*Fusarium* sp FAN2011a [79%]	*Fusarium bataticola* [97%]
65	Pleosporaceae sp [100%]	*Alternaria* sp3 MU2012 [100%]	Pleosporales sp. [100%]
76	Ascomycota sp [52%]	*Fusarium* sp FAN2011e [75%]	*Fusarium acuminatum* [99.91%]
103	Ascomycota sp [43%]	*Fusarium* sp FAN2011a [68%]	*Fusarium irregulare* [99.82%]
130	Nectriaceae sp [61%]	*Fusarium* sp FAN2011a [75%]	*Fusarium oxysporum* [99.82%]
144	Xylariales sp [100%]	*Pestalotiopsis* sp7 MU2012 [61%]	*Microdochium bolleyi* [99.36%]
S1	Dothideomycetes sp [73%]	*Periconia* sp9 MU2012 [92%]	Pleosporales sp. [98.22%]
S2	Hypocreales sp [90%]	*Fusarium* sp FAN2011a [81%]	*Sarocladium strictum* [92.65%]
S3	*Rhizochaete filamentosa* [74%]	*Phanerochaete magnoliae* [40%]	*Porostereum crassum* [98.70%]
S4	Xylariales sp [100%]	*Microdochium bolleyi* [76%]	*Microdochium bolleyi* [94.59%]
S5	*Podospora glutinans* [99%]	*Fusarium* sp FAN2011a [77%]	*Schizothecium curvisporum* [97.99%]
S6	Xylariales sp [100%]	*Microdochium bolleyi* [47%]	*Microdochium bolleyi* [95.67%]
S7	Helotiales sp [41%]	*Leveillula lanuginose* [52%]	Uncultured fungus [94.56%]
S8	Diatrypaceae sp [54%]	*Nigrospora* sp5 MU2012 [66%]	Xylariales sp. [97.54%]

S9	Ascomycota sp [100%]	*Leveillula lanuginose* [56%]	uncultured fungus [99.82%]
S10	Ascomycota sp [100%]	uncultured Protoventuria [87%]	Uncultured *Leptodontidium* [99.32%]
S11	Hypocreales sp [83%]	*Fusarium* sp FAN2011a [74%]	*Hydropisphaera* sp. [99.47%]

Table S2. Pairwise comparisons of community composition (PERMANOVA) and heterogeneity (PERMDISP) between N-addition treatments within each *Epichloë* treatment.

**Within E- Treatments**	**Composition**		**Heterogeneity**	
**N Addition Comparison**	**t**	***P***	**t**	***P***
low vs. high	0.91	0.514	0.18	0.872
low vs. none	0.97	0.521	2.72	**0.023**
high vs. none	1.15	0.275	2.36	**0.052**

**Within E+ Treatments**	**Composition**		**Heterogeneity**	
**N Addition Comparison**	**t**	***P***	**t**	***P***
low vs. high	1.71	**0.032**	0.58	0.606
low vs. none	1.62	**0.040**	1.22	0.242
high vs. none	2.43	**0.001**	0.54	0.598

Table S3. Similarity Percentages (SIMPER) Analysis results, showing taxon contributions to community composition similarity within each *Epichloë* × N addition treatment, up to 90% cumulative composition.

E-, control N

OTU	Likely ID	Ave. Frequency	% Contribution	Cumulative %
OTU31	*Microdochium bolleyi*	0.8	53.24	53.24
OTU18	*Fusarium fujikuroi*	0.6	27.85	81.09
OTU12	*Sarocladium strictum*	0.3	5.49	86.57
OTU6	*Penicillium bilaiae*	0.3	4.8	91.37

E-, low N

OTU	Likely ID	Ave. Abundance	% Contribution	Cumulative %
OTU31	*Microdochium bolleyi*	1	62.29	62.29
OTU18	*Fusarium fujikuroi*	0.7	24.8	87.09
OTU16	*Leptosphaeria* sp	0.5	11.84	98.92

E-, high N

OTU	Likely ID	Ave. Abundance	% Contribution	Cumulative %
OTU18	*Fusarium fujikuroi*	0.9	41.74	41.74
OTU31	*Microdochium bolleyi*	0.9	39.99	81.74
OTU16	*Leptosphaeria* sp	0.5	10.51	92.25

E+, control N

OTU	Likely ID	Ave. Abundance	% Contribution	Cumulative %
OTU18	*Fusarium fujikuroi*	1	49.11	49.11
OTU31	*Microdochium bolleyi*	0.9	34.89	84
OTU16	*Leptosphaeria* sp	0.6	12.16	96.16

E+, low N

OTU	Likely ID	Ave. Abundance	% Contribution	Cumulative %
OTU31	*Microdochium bolleyi*	1	56.18	56.18
OTU18	*Fusarium fujikuroi*	0.67	19.75	75.93
OTU16	*Leptosphaeria* sp	0.56	14.87	90.8

E+, high N

OTU	Likely ID	Ave. Abundance	% Contribution	Cumulative %
OTU31	*Microdochium bolleyi*	0.9	31.68	31.68
OTU18	*Fusarium fujikuroi*	0.8	23.44	55.12
OTU57	*Fusarium sporotrichioides*	0.7	19.76	74.88
OTU7	*Acremonium* sp.	0.7	17.85	92.73

Table S4. Similarity Percentages (SIMPER) Analysis results, showing root endophyte taxon average frequencies and their contribution to community differences among N addition treatments when *Epichloë* was present.

Control vs. Low N: average dissimilarity = 46.33

	***Leptosphaeria* sp.**	***Fusarium* sp.**	***Fusarium* sp.**	***Sarocladium strictum***	***Cadophora* sp.**	***Penicillium bilaiae***
Control	0.60	0	1.00	0.30	0.20	0.20
Low N	0.56	0.44	0.67	0.11	0.11	0.11
Dissimilarity contrib. %	15.99	13.45	12.68	9.02	7.85	7.05

Control vs. High N: average dissimilarity = 51.06

	***Fusarium* sp.**	***Uncultured Acremonium***	***Leptosphaeria* sp.**	***Sarocladium strictum***	***Penicillium bilaiae***	***Fusarium* sp.**
Control	0	0.10	0.60	0.30	0.20	1.00
High N	0.70	0.70	0.40	0.10	0.20	0.80
Dissimilarity contrib. %	18.54	16.93	13.15	7.48	7.34	6.32

Low vs. High N: average dissimilarity = 50.72

	Uncultured Acremonium	***Fusarium* sp.**	***Leptosphaeria* sp.**	***Fusarium* sp.**	***Penicillium bilaiae***	***Fusarium* sp.**
Low N	0	0.44	0.56	0.67	0.11	0.11
High N	0.70	0.70	0.40	0.80	0.20	0.20
Dissimilarity contrib.%	17.08	13.54	12.92	11.04	5.69	5.69

Figure S1. Heatmap displaying presence/absence of fungal root endophyte OTUs within each *Epichloë* and N addition treatment

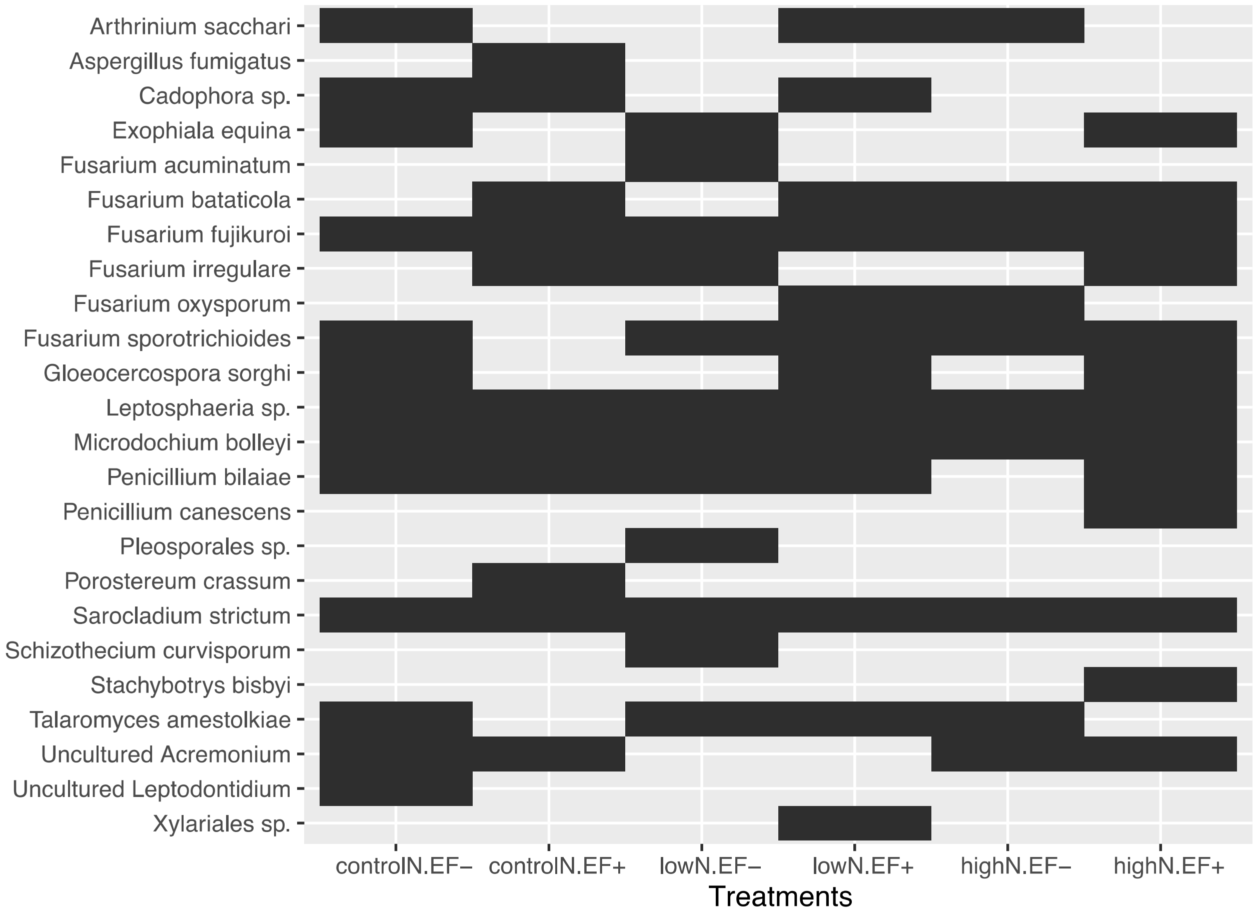

Supplement: Supplementary file 1 [file jof-08-01142-s001.zip › jof-1974607-supplementary.pdf]
